# Research Progress on the Relationship Between PRPF8 and Cancer

**DOI:** 10.3390/cimb47030150

**Published:** 2025-02-26

**Authors:** Guoqing Huang, Dandan Wang, Jiaying Xue

**Affiliations:** Institute of Advanced Technology, Heilongjiang Academy of Sciences, Harbin 150001, China

**Keywords:** PRPF8, alternative splicing, cancer

## Abstract

Alternative splicing (AS) plays a crucial role in regulating gene expression and protein diversity, influencing both normal cellular function and pathological conditions, including cancer. Protein pre-mRNA processing factor 8 (PRPF8), a core component of the spliceosome, is integral to the splicing process, ensuring accurate gene transcription and spliceosome assembly. Disruptions in PRPF8 function are linked to a variety of cancers, as mutations in this gene can induce abnormal splicing events that contribute to tumorigenesis, metastasis, and drug resistance. This review provides an in-depth analysis of the mechanisms by which PRPF8 regulates tumorigenesis through AS, exploring its role in diverse cancer types, including breast, liver, myeloid, and colorectal cancers. Furthermore, we examine the molecular pathways associated with PRPF8 dysregulation and their impact on cancer progression. We also discuss the emerging potential of targeting PRPF8 in cancer therapy, highlighting challenges in drug development.

## 1. Introduction

AS is a crucial factor in the complexity of transcriptional and protein-level regulation in multicellular eukaryotes, significantly influencing both physiological and pathological conditions in organisms [1]. AS regulates a wide array of highly diverse biological processes, including tissue specificity, cell differentiation, thermoregulation, infrared sensing, the Warburg effect, telomere length maintenance, and cancer [2]. PRPF8, a core protein in the AS process, plays an essential role. Numerous studies have demonstrated that when PRPF8 function is disrupted, a significant number of alternative splicing events, such as intron retention and exon skipping, occur. The production of loss-of-function proteins becomes a key factor in the onset and progression of various cancers [3,4,5,6]. Therefore, this paper summarizes the mechanisms through which PRPF8 regulates tumorigenesis, reviews the impact of PRPF8 expression on various cancers, and discusses its potential clinical applications. 

## 2. Overview of PRPF8 

AS is a fundamental cellular process in which the spliceosome removes introns from precursor mRNA and subsequently rearranges and joins exons to form mature mRNA [7]. High-throughput sequencing has revealed that over 95% of genes in the human genome undergo AS, highlighting its critical role in cellular function [8]. A single gene can give rise to multiple proteins with distinct structures and functions, significantly contributing to protein diversity. This phenomenon may be a key factor explaining why humans, despite having a similar number of genes to other species, possess more complex protein functions and regulatory mechanisms. The spliceosome, which consists of five subcomplexes of small nuclear ribonucleoproteins (snRNPs)—U1, U2, U4, U5, and U6—along with over 200 associated factors, plays a crucial role in the AS process [9]. Disruption of the spliceosome’s cutting and splicing functions can alter the original mRNA coding sequence, potentially leading to the production of isoforms with distinct cellular functions or characteristics, thereby impacting nearly every biological process. During mRNA splicing, U1 snRNP first recognizes the 5′ splice site (5′SS), followed by U2 snRNP’s recognition of the branch point and the 3′ splice site (3′SS), completing the assembly of the spliceosome on the pre-mRNA (spliceosome complex A). Concurrently, U4, U5, and U6 snRNPs form a complex (U4/U6-U5 tri-snRNP), which then associates with U1 and U2 to form spliceosome complex B. Subsequently, U1 and U4 snRNPs detach, and U2, U5, and U6 snRNPs remain to form spliceosome complex C, where they catalyze two transesterification reactions to excise the intron from the pre-mRNA [10].

### 2.1. The Function of PRPF8

**Figure 1:**
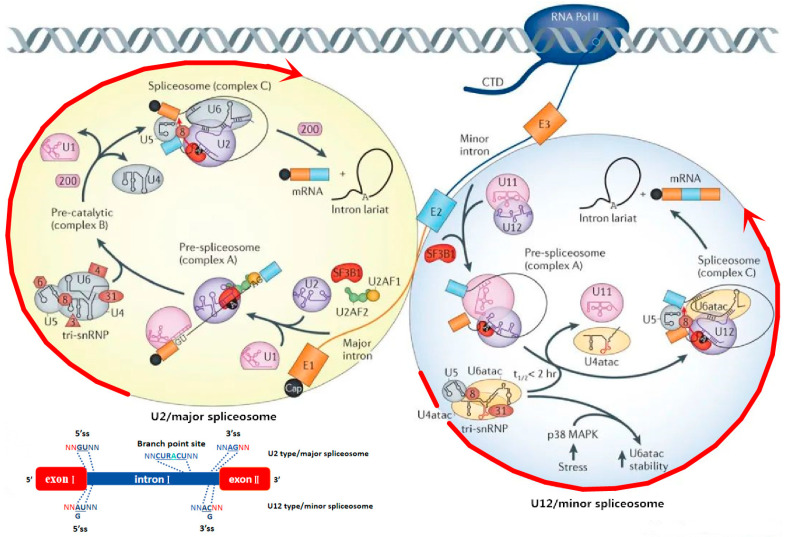
The splicing process of U2- and U12-dependent spliceosome [13]. Note: The steps surrounded by the long red arrows are PRPF8 participating steps.

PRPF8, a critical component of the U5 snRNP, is located on chromosome 17p13.3 in human cells and is encoded by 43 exons, with a molecular weight of approximately 220 kDa. As the largest protein in the spliceosome, it serves as a core element of the pre-catalytic, catalytic, and post-catalytic spliceosome complexes, playing an indispensable role in pre-mRNA splicing [11,12]. PRPF8 is involved in both the U2-dependent spliceosome (major spliceosome) and the U12-dependent spliceosome (minor spliceosome), contributing significantly to the catalytic process of pre-mRNA splicing, as depicted in Figure 1 [13]. In both yeast and human cells, PRPF8 interacts with the 5′SS, the branch point, the polypyrimidine tract, and the 3′SS during splicing [14,15,16]. PRPF8 also facilitates the folding of the U2/U6 catalytic pre-mRNA complex [17]. Moreover, it is found within the human spliceosome in association with RBM22 and SLU7 [18,19]. Acting as a scaffold protein, PRPF8 positions the spliceosome subunits U2, U5, and U6-snRNP at the pre-mRNA splice sites, promoting the orderly assembly of the U4/U6-U5 tri-snRNP complex and the snRNAs [20,21,22]. Additionally, PRPF8 regulates several splicing-related factors, including EFTUD2 and SNRNP200, thus maintaining the accuracy of gene transcription and regulating cellular functions [10]. Furthermore, PRPF8 can specifically impact spliceosome assembly by inhibiting its formation, which reduces the number of fully assembled functional spliceosomes, ultimately leading to splicing abnormalities [23]. In this context, PRPF8 plays a crucial role in ensuring the precision and efficiency of the entire splicing process [24,25,26,27]. The absence of PRPF8 is associated with the development of various physiological and pathological diseases [28,29,30]. 

### 2.2. Disorder of PRPF8

The spliceosome, which consists of several protein and RNA components, catalyzes the removal of introns and the joining of exons to produce mature mRNA. PRPF8 is a crucial player in this process, ensuring the accuracy and efficiency of splicing. In cancer cells, however, the function of PRPF8 may be altered due to mutations, leading to the production of abnormal splicing isoforms of genes that regulate critical pathways. It is currently estimated that each human protein-coding gene encodes an average of 7.4 RNA isoforms [31]. The spliceosome plays a crucial role in various biological processes, including cancer. A pan-cancer analysis revealed the alteration landscape of spliceosome genes across 27 cancer types in 9070 patients, demonstrating both common and specific changes in spliceosome genes among different cancer types, with PRPF8 showing a high mutation rate (7%) [32]. Although missense mutations in PRPF8 are distributed across the entire gene, the vast majority of these mutations do not significantly affect the biological function of PRPF8, indicating that the biological functions of the PRPF8 protein are relatively conserved [22]. Most mutations that alter the biological function of PRPF8 are concentrated in the C-terminal Jab1/MPN domain, which interacts with SNRNP200 [22]. Neither wild-type nor mutant PRPF8 (as observed in retinitis pigmentosa) affected cell proliferation. Both wild-type and all mutated forms of PRPF8 localized to the cell nucleus, but most mutated proteins were also partially present in the cytoplasm [33,34]. Additionally, the degradation rate of most PRPF8 mutant proteins was faster than that of the wild-type PRPF8 [33,34]. Among these, the prognosis for mutant PRPF8 was better than that for wild-type PRPF8 in retinitis pigmentosa [34]. 

In cells lacking PRPF8 function, there is a reduction in spliceosome assembly, decreased splicing efficiency, and aberrant AS events [35]. Mutations in PRP8 (the yeast homolog of PRPF8) also lead to reduced assembly efficiency of the U5 snRNP, causing disruptions between the first and second steps of AS. The interaction efficiency of PRPF8 with splicing factors (SFs) Snu114 and Brr2 is reduced, and it interferes with the helicase activity regulated by Brr2, ultimately leading to splicing errors in pre-mRNA and resulting in incomplete and inconsistent growth defects in yeast [20,35,36,37]. In contrast, the PRPF8 mutations p.Tyr334Asn and p.Phe2314Leu do not affect spliceosome assembly. However, these mutations inhibit the helicase activity of SNRNP200 and weaken its association with SNRNP200, leading to regulatory errors in SNRNP200, which may contribute to splicing abnormalities [24]. 

An increasing number of studies confirm that alterations in the AS process mediated by PRPF8 are associated with various cancers. Pro-tumorigenic AS is now considered a hallmark of cancer [38,39,40]. However, the precise mechanisms by which PRPF8 mutations affect the overall functionality of spliceosome proteins and contribute to malignant phenotypes remain unclear. Additionally, it is not fully understood how these mutations result in distinctly different disease manifestations.

## 3. PRPF8 Mutations Across Cancer Types

The vast majority of AS isoforms do not significantly impact normal cellular function, and most abnormal isoforms are cleared by the human proofreading and metabolic mechanisms. However, RNA splicing dysregulation, such as spliceosome mutations, changes in the expression levels of components involved in the splicing mechanism, alterations in SF levels or activity, or other factors, may disrupt gene product networks and affect cancer pathways [41,42]. RNA splicing dysregulation is a molecular hallmark of nearly all cancer types [22,43]. Tumors typically express more complex SFs than normal tissues, and tumorigenicity may be linked to cancer-specific AS events that arise during the transformation process [42]. Cancer-associated AS isoforms affect thousands of genes, providing cells with proliferative advantages, enhancing cell migration and metastasis, protecting cells from death, reprogramming cell metabolism or signaling, promoting the tumor-supportive microenvironment, altering immune responses, or inducing drug resistance [42,44,45]. Functional studies in model systems suggest that different stages of tumorigenesis may require combinations of multiple AS isoform switches to be completed [33,42,46,47]. Recurrent SF mutations are common in hematologic malignancies, while changes in SF levels and copy number alterations are particularly prominent in solid tumors [48]. 

PRPF8, as a core component of the AS process, has been extensively studied since 2005, primarily as a factor associated with retinal pigmentary degeneration [4,5,21]. More recently, studies have shown that somatic mutations in PRPF8 are closely linked to various types of cancer, including breast cancer, liver cancer, colorectal cancer, and lung cancer. These recurrent somatic mutations lead to incorrect splicing of transcripts, thereby promoting cancer growth and progression. 

### 3.1. Breast Cancer 

Cortes-Urrea et al. analyzed exome sequencing data and found that PRPF8 gene mutations are tumor-driving mutations in breast cancer [49]. Studies have also indicated that knockdown of PRPF8 in various types of breast cancer cells leads to increased intron retention, AS isoforms, and cell apoptosis, as well as affecting cellular protein metabolism, mitosis, and proteasome function [50,51]. However, the function of PRPF8 is not entirely consistent across different breast cancer subtypes, demonstrating subtype specificity [52]. Furthermore, the study by Cao et al. showed that knockdown of PRPF8 significantly inhibits the growth of breast cancer cells and weakens their clonogenic ability [53]. These studies all suggest a close relationship between PRPF8 and breast cancer. Additionally, through analysis of TCGA and the Kaplan–Meier Plotter database, we found that breast cancer patients with high PRPF8 expression have a shorter disease-free survival and poorer prognosis, further emphasizing the importance of in-depth research on the relationship between PRPF8 and breast cancer for clinical applications. 

### 3.2. Myeloid Tumor

Based on cellular morphology, immunophenotype, molecular genetics, and clinical characteristics, the World Health Organization classifies myeloid tumors into three categories: Myelodysplastic Syndromes (MDS), Myeloproliferative Neoplasms (MPN), and Acute Myeloid Leukemia (AML) [54]. Studies have shown that functional loss caused by mutations and del(17p) in PRPF8 is closely associated with poor prognosis in myeloid tumor patients [55]. A large cohort analysis revealed that PRPF8 mutations frequently occur in myeloid malignancies, primarily including missense mutations, nonsense mutations, frameshift mutations, and splice site mutations [56]. Moreover, the degradation rate of most PRPF8 mutant proteins is faster than that of wild-type PRPF8 [34,56]. In myeloid tumors, most PRPF8 mutations are located in exon 42, with a few mutations (including p.Ser2118Phe) found in exon 38 [57,58,59,60]. The frequency of somatic PRPF8 mutations in patients with MDS, primary and secondary AML, and different MPN subtypes is 4% (65/1700), 4–5%, and 10%, respectively. Furthermore, over 50% of PRPF8-mutated AML patients have a poor prognosis [55,56]. This suggests that, compared to low-risk MDS, PRPF8 mutations are associated with more aggressive cancer phenotypes. Additionally, Růžičková et al. found that PRPF8 mutations led to increased in aggressive myeloid malignancies, with a higher presence of ringed sideroblasts [20]. Furthermore, some studies indicate that PRPF8 splicing dysregulation is related to increased myeloid cells, although no reports have confirmed its specific role in myeloid malignancies [61,62]. 

### 3.3. The Other Cancers

In liver cancer cells, PRPF8 expression is abnormally elevated and plays a crucial role in liver carcinogenesis by altering FN1 splicing, activating the FAK/AKT pathway, and promoting stress fiber formation. PRPF8 expression is also associated with the invasiveness of liver cancer cells [63]. Furthermore, upregulation of PRPF8 correlates with poor prognosis in clinical liver cancer patients, acting as a tumor promoter by regulating the PI3K/Akt pathway in hepatic stellate cells and HCC cells, thereby enhancing liver cancer cell viability and metastatic potential. Moreover, the loss of PRPF8 significantly inhibits the malignancy of liver cancer cells [64]. In prostate cancer, PRPF8 functions as a novel cofactor for the androgen receptor. PRPF8 interacts with the androgen receptor, regulating its function in prostate cancer cells and controlling its transcriptional activity. Additionally, knockdown of PRPF8 increases the polyubiquitination of endogenous androgen receptors [65], which could serve as a potential mechanism through which PRPF8 regulates androgen receptor activity. In colorectal cancer, Abdel-Wahab et al. demonstrated that silencing PRPF8 in DLD1 colon cancer cells led to cell death, indicating that PRPF8 regulates both cell growth and apoptosis in colorectal cancer [51]. Moreover, Zhang et al. confirmed that PRPF8 mediates the splicing of PRMT7-V2 [66]. Overexpression of PRMT7-V2 significantly promotes the growth of colorectal cancer cells and xenograft tumors, with the selective PRMT7 inhibitor SGC3027 exerting antitumor effects on human colorectal cancer cells. In lung cancer, a significant difference in PRPF8 expression is observed between cancerous and adjacent tissues, with PRPF8 expression significantly affecting lung cancer cell proliferation and colony formation [67]. Additionally, analysis using the ENCORI database and related studies revealed that the stress response of lung cancer cells to chemotherapy is mediated by a reduction in selective splicing events, including those involving PRPF8, leading to decreased gene transcription [68]. These findings offer new insights into the molecular mechanisms underlying cancer cell dormancy and reactivation. In ovarian cancer, PRPF8 expression significantly promotes ovarian cancer cell proliferation and inhibits apoptosis through the circRNA-UBAP2/miR-382-5p/PRPF8 pathway [69]. In pancreatic intraductal papillary mucinous neoplasms, specific gene mutations are rare; however, rare PRPF8 mutations were identified through whole-exome sequencing of primary ductal papillary mucinous neoplasm tissues [70]. 

## 4. Mechanisms by Which PRPF8 Regulates

PRPF8 is an essential component of the spliceosome, playing a crucial role in its assembly process and ensuring the accuracy of alternative splicing. When PRPF8 is disordered in spliceosome assembly, it promotes tumorigenesis [21,22]. PRPF8 mutations can also alter the internal dynamics of the spliceosome, modifying the splicing pattern and causes global changes in splice site recognition and exon inclusion that particularly affect genes involved in these cellular functions [61,71,72]. Functional disruption of PRPF8 not only affects tumor growth and metastasis but also enhances tumor invasiveness and metastasis by modulating key signaling pathways, such as the FAK/AKT, PI3K/Akt, and p53 pathways, and their critical factors [5,63,64]. PRPF8 increases the aggressiveness of hepatocellular carcinoma by regulating the FAK/AKT pathway via fibronectin 1 splicing [5]. The PI3K/Akt pathway is essential for regulating cell survival, proliferation, and metabolism. Abnormal splicing events in PRPF8 can affect the expression of components of the PI3K/Akt pathway, leading to constitutive activation of Akt, which promotes tumorigenesis and resistance to chemotherapy [64]. As a critical tumor suppressor, p53 regulates cell cycle checkpoints, apoptosis, and DNA repair. PRPF8 can alter the splicing of p53 isoforms through PRPF8–PIRH2–p53 axis, which can impair its ability to suppress tumor growth effectively [63]. These signaling pathways are well known for their crucial roles in tumor cell proliferation, migration, invasion, and drug resistance. Additionally, Wood et al. suggested that cancer-related variants may affect the entire length of the PRPF8 protein, thereby influencing its diverse functions and/or interactions [22]. It is essential to further investigate how specific mutations in PRPF8 affect AS in a context-dependent manner, particularly in relation to tumor type and stage. The identification of "hot spots" within the PRPF8 gene that are frequently mutated across cancer types could offer insights into therapeutic strategies aimed at restoring proper splicing patterns. 

## 5. Preclinical Drug Research Targeting PRPF8

As the potential of PRPF8 in cancer treatment becomes increasingly apparent, targeted drug research focusing on PRPF8 has emerged as a key area of interest in cancer therapy. The PRPF8-dependent apoptosis pathway is believed to offer therapeutic benefits in various cancer types, particularly in tumors that exhibit resistance to conventional treatments such as chemotherapy or radiotherapy [63]. As outlined in Table 1, several candidate drugs targeting PRPF8 have entered the preclinical research phase. However, there are several challenges currently hindering progress in this field: (1) Drug targeting and selectivity: Given that PRPF8 is a complex molecule with multiple functions, developing drugs with high targeting specificity and minimal off-target effects on normal cells presents a significant technical challenge. (2) Pharmacokinetics of the drugs: Ensuring the effective delivery of the drug to the tumor site and achieving a sufficient concentration at the target location is another crucial hurdle in the development of PRPF8-targeted drugs. (3) Relevance and translatability of animal models: Although in vitro data are relatively abundant, the ability of various animal models to accurately reflect clinical responses in human cancers requires further validation. Despite these challenges, the promising results of PRPF8-targeted drugs in the experimental stage (see Table 1) suggest potential, though the transition from in vitro research to clinical application will require overcoming many technical and clinical barriers. This underscores the need for further in vivo studies, optimization of the drug, and accumulation of clinical trial data before PRPF8-targeted therapies can achieve widespread clinical application. 

Preclinical drug research targeting PRPF8 remains in its early stages. While positive results have been observed in laboratory settings, the true clinical value of these therapies must be validated through additional animal models and preclinical studies. The therapeutic potential of the PRPF8-dependent apoptosis pathway warrants further investigation. Nevertheless, the journey from basic research to clinical application will require substantial time and effort for validation and refinement.

## 6. Discussion

Although mutations in the *PRPF8* gene are frequently observed in various cancers through TCGA database analyses, the mechanisms underlying the splicing errors in the diverse gene subgroups regulated by PRPF8 remain unclear. Additionally, the reasons why different PRPF8 mutations and/or varying expression levels contribute to distinct diseases, such as various cancers or retinal diseases, remain elusive. Nonetheless, these observations suggest that cells are highly sensitive to PRPF8 expression levels and function. Any deviations in these factors can significantly impact the phenotype of specific cancers and/or diseases. The direction and magnitude of these deviations may play a crucial role in influencing the phenotypes of various diseases, including cancer, in patients. We hypothesize that different types of PRPF8 mutations, or mutations affecting distinct functional domains of PRPF8, may alter the interactions between the spliceosome and pre-mRNA, including catalytic functions. However, further evidence is needed to substantiate this hypothesis. The molecular regulatory mechanisms and key targets of PRPF8 remain poorly understood, and to fully establish its clinical relevance, more in vivo and in vitro experimental data are required. 

### 6.1. Key Regulatory Target Screening and Regulation Methods Require In-Depth Research

Numerous studies have demonstrated that PRPF8 regulates AS of various genes, either directly or indirectly, across different tumors, thereby significantly influencing apoptosis in tumor cells. However, the regulatory factors reported in these studies are inconsistent, and downstream targets of PRPF8, such as PIRH2, RBMX, and APC2, exhibit low reproducibility across different tumor types [63,73,75]. Therefore, further investigation into the widely applicable downstream targets activated by PRPF8 across different tumors or at various stages of tumor progression may enhance the understanding of cancer development mechanisms and offer promising implications for cancer therapy. Additionally, several important questions regarding the role of PRPF8 in tumors remain unresolved, including how these regulatory molecules interact, which molecule plays a decisive role, and whether the evolving regulatory network is accurate. These questions have yet to be addressed in the literature. 

The development of therapies aimed at modulating PRPF8 function is an exciting area of research. The small molecule inhibitors such as SGC3027 and pladienolide B have shown promise in targeting the spliceosome [40,66]. In addition, antisense oligonucleotides (ASOs) also could be used to specifically modulate the splicing of PRPF8 itself or target the splicing of downstream genes affected by PRPF8 mutations. By correcting aberrant splicing at the RNA level, ASOs may serve as a potent therapeutic strategy, although challenges remain in designing ASOs with high specificity and low off-target effects. 

Moreover, to address these challenges, next-generation sequencing and RNA-Seq could be utilized to identify the full spectrum of splicing alterations caused by PRPF8 mutations in various cancer types will reveal novel biomarkers and therapeutic targets. Identifying specific splicing alterations in tumorigenesis driven by PRPF8 mutations could serve as biomarkers for early diagnosis and prediction of therapeutic responses. Furthermore, more robust preclinical models, including patient-derived xenografts and organoids, should be used to evaluate the efficacy of PRPF8-targeted therapies in vivo in next stages. 

### 6.2. Limitations of the Pathways

Tumor therapies targeting PRPF8 from various angles and multiple pathways are actively being researched and developed; however, the current body of research remains largely theoretical. Pathways involving PRPF8, such as the PRPF8–PIRH2–p53 pathway [63], have notable limitations, with their effects primarily confined to specific tumor types, and the efficacy of different pathways varies significantly across tumors. To date, there are no widely recognized findings on signaling pathways that comprehensively integrate the functions of PRPF8, which severely limits the clinical applicability of PRPF8-targeted therapies. Moreover, the mechanisms by which PRPF8 exerts both supportive and inhibitory effects in tumors remain unclear, and no systematic regulatory network has been fully delineated. Mutations and/or alterations in the expression of PRPF8 proteins in cancers have been implicated in the dysregulation of pre-mRNA splicing, thereby promoting tumorigenesis. However, the mechanisms through which different mutations and/or varying expression levels of the same PRPF8 gene lead to contrasting phenotypes, such as retinal or craniofacial defects and cancer, remain enigmatic. In particular, the precise mechanisms by which distinct subsets of PRPF8 undergo mis-splicing in these contexts remain unclear. It is possible that variants affecting different functional domains of PRPF8 may alter its interactions with specific classes of pre-mRNAs, depending on their characteristics, though additional evidence is needed to substantiate this hypothesis [16]. Nonetheless, these findings suggest that human cells are highly sensitive to PRPF8 expression levels and function, with even minor deviations potentially resulting in significant cancer-related consequences. The direction and magnitude of these deviations likely play a critical role in determining the phenotypic outcomes in patients.

## 7. Conclusions

In conclusion, PRPF8 is an essential regulator of alternative splicing, and its dysregulation is closely tied to various aspects of tumorigenesis. Understanding the molecular mechanisms by which PRPF8 mutations contribute to cancer progression will provide valuable insights into the development of novel cancer therapies. However, challenges such as the complexity of spliceosome function, the need for precise targeting, and the avoidance of off-target effects remain significant hurdles. Future research focusing on the precise mechanisms of PRPF8 in tumorigenesis, coupled with the development of targeted therapeutic approaches, will provide a promising avenue for personalized cancer treatments. 

## Figures and Tables

**Table 1:** The study of preclinical tumor model targeting PRPF8.

Cancer Type	Research Model	Study Drugs
Colorectal cancer	Cells, xenografts	PRMT7 inhibitor (SGC3027) [66]
Breast cancer	Cells	PRPF8 inhibitor [53]
Ovarian cancer	Cells	circRNA-UBAP2 inhibitor [69]
Pancreatic cancer	Cells	PRPF8 and RBMX [73]
Hepatocellular carcinoma	Cells, xenografts	fibronectin 1 [5]
Osteosarcoma	Cells	Pladienolide B [40]
HER2-positive breast cancer	Cells	ZNF649-AS1 [74]
Prostate cancer	Cells	PRPF8 and androgen receptor [65]
Cervical cancer	Cells	Sororin and APC2 [75]
